# Association of atherogenic index of plasma with urine albumin-to-creatinine ratio in Chinese urban adults: a cross-sectional study

**DOI:** 10.1080/0886022X.2026.2657102

**Published:** 2026-06-01

**Authors:** Yu Hou, Senyuan Chen, Qingzheng Wu, Yue Zhang, Haiming Wang, Yu Cheng, Bing Li, Yiming Mu

**Affiliations:** a Chinese PLA Medical School, Beijing, China; b Department of Endocrinology, The First Medical Center of People’s Liberation Army General Hospital, Beijing, China; c Sichuan Corps of People’s Armed Police Force, Chengdu, China

**Keywords:** Atherogenic index of plasma, urine albumin-to-creatinine ratio, chronic kidney disease, albuminuria

## Abstract

The association between the atherogenic index of plasma (AIP) and urine albumin-to-creatinine ratio (UACR) has not been fully elucidated in large-scale populations. This study aimed to systematically examine the association between AIP and elevated UACR in Chinese urban adults. UACR was categorized as normal (UACR < 30 mg/g) and albuminuria (UACR ≥ 30 mg/g), and participants were divided into quartiles according to AIP distribution. Multivariable logistic regression models were used to evaluate the association between AIP and elevated UACR after adjustment for potential confounders. Higher AIP quartiles were significantly and positively associated with increased odds of albuminuria. In the fully adjusted model, the odds ratios for elevated UACR were 1.304 (95% CI: 1.196, 1.422), 1.475 (95% CI: 1.355, 1.607), and 1.815 (95% CI: 1.668, 1.975) for the second, third, and fourth AIP quartiles, respectively, compared with the lowest quartile (all *p* < 0.001). Subgroup analyses indicated that this association was more pronounced among individuals younger than 60 years and those not using lipid-lowering medications. These findings suggest that elevated AIP level is independently associated with increased UACR in the Chinese urban population, particularly in younger individuals not receiving lipid-lowering therapy.

## Introduction

Chronic kidney disease (CKD) has been severely threatening global public health as a chronic non-communicable disease. With the constantly elevating incidence, CKD is causing a profound impact on healthcare burden and patients’ quality of life [1]. According to GBD 2023 estimates, CKD is now the ninth leading cause of death globally, affecting approximately 788 million people aged ≥20 years worldwide [2]. The urine albumin-to-creatinine ratio (UACR), a key biomarker for early glomerular injury, is widely used in clinical practice for initial screening, disease monitoring, and prognosis assessment due to its ease of testing and high reproducibility [3]

As CKD progresses, significant alterations occur in patients’ lipid profiles, including their levels, composition, and metabolic characteristics. These changes further increase the risk of atherosclerosis [4]. The typical lipid profile in CKD is marked by hypertriglyceridemia, reduced high density lipoprotein cholesterol (HDL-C), elevated low density lipoprotein cholesterol (LDL-C), and fluctuations in total cholesterol (TC) [4–6]. A cross-sectional study in Korea showed that UACR is positively correlated with the levels of TC and triglyceride (TG), and is inversely correlated with HDL-C [7]. Furthermore, our previous study conducted in Chinese adult population also demonstrated a high correlation between UACR and residual cholesterol [8].

The atherogenic index of plasma (AIP), a novel index for identifying dyslipidemia and insulin resistance, is an established independent predictor of cardiovascular diseases [9,10]. It is calculated as the logarithm of the ratio of TG to HDL-C [11]. Previous research has shown that elevated AIP levels are significantly associated with an increased incidence of cardiorenal syndrome [12]. Evidence from another retrospective study showed that AIP has a strong positive correlation with UACR in a population of 600 youths with type 1 diabetes mellitus [13]. Although a few studies have suggested a potential correlation between AIP and UACR, a systematic investigation based on a large-scale population is still needed. Therefore, this study collected clinical and laboratory data from 49,799 Chinese urban adults to investigate the correlation between AIP and UACR, providing a basis for further prospective validation.

## Methods

### Participants and study design

Population of this work was derived from the Risk Evaluation of Cancers in Chinese Diabetic Individuals (REACTION) study, which was designed to research the association between diabetes or prediabetes and the risk of cancer in Chinese population [14]. The REACTION study enrolled 53,639 participants from eight centers across China (Dalian, Lanzhou, Zhengzhou, Guangzhou, Guangxi, Luzhou, Shanghai, and Wuhan) between 2011 and 2012. All participants are permanent adult residents aged 40 or above from 3 to 5 communities randomly elected from the city, and all the participants have signed the informed consent. The research program was authorized by the Human Research of Rui-Jin Hospital affiliated with the School of Medicine of Shanghai Jiao Tong University.

**Figure 1.**
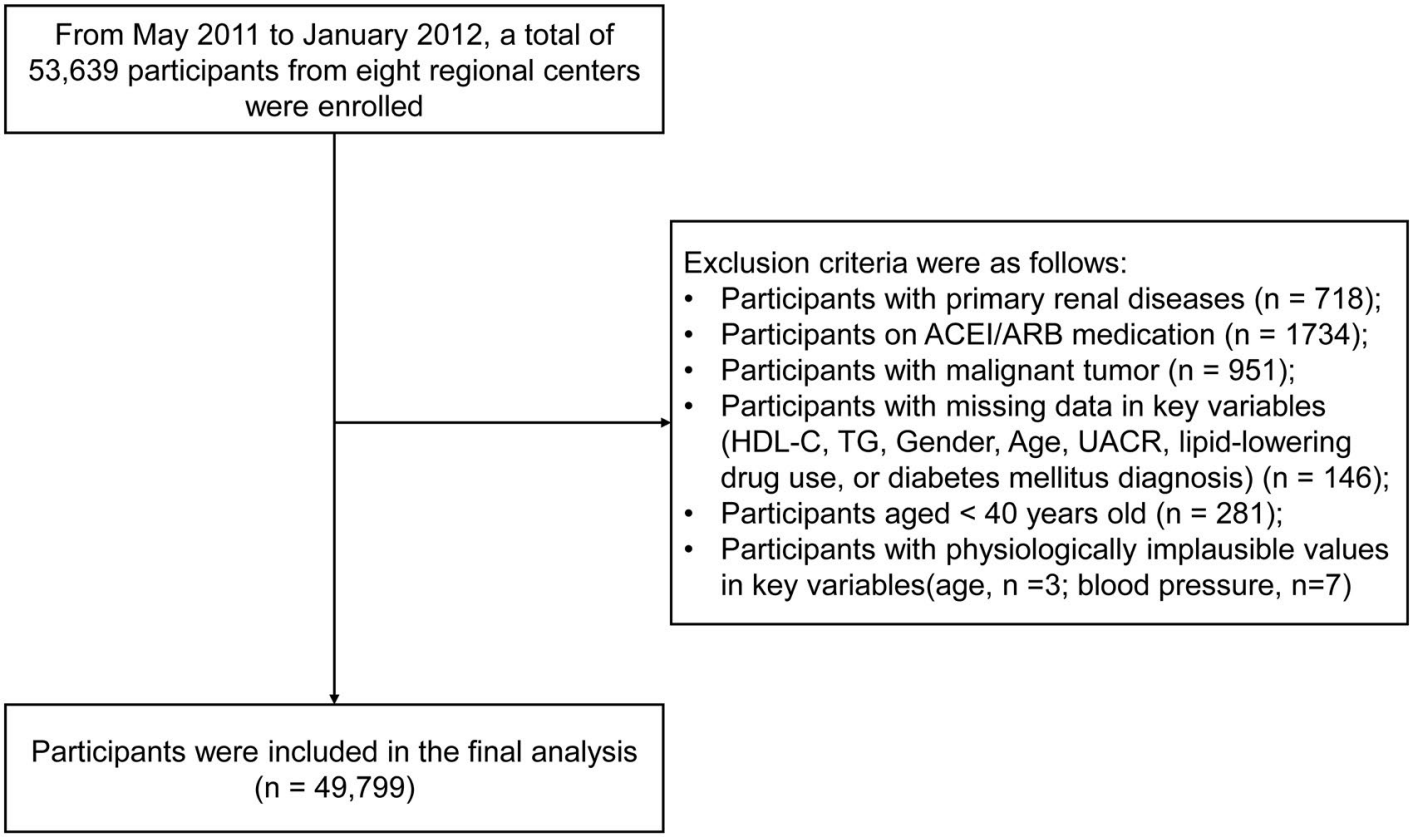
Flow diagram of participant enrollment and selection. The flowchart illustrates the sequential inclusion and exclusion criteria applied to the initial cohort to derive the final sample of 49,799 participants used for the primary analysis. ACEI: Angiotensin-Converting Enzyme Inhibitor; ARB: Angiotensin II Receptor Blocker; HDL-C: High-Density Lipoprotein Cholesterol; TG: Triglycerides; UACR: Urinary Albumin-to-Creatinine Ratio.

Exclusion criteria: Participants with primary renal diseases (*n* = 718); Participants on ACEI/ARB medication (*n* = 1734); Participants with malignant tumor (*n* = 951); Participants with missing data in key variables (HDL-C, TG, sex, age, UACR, lipid-lowering drug use, or diabetes mellitus diagnosis)(*n* = 146); Participants aged <40 years old (*n* = 281); Participants with physiologically impossible values (age, *n* = 3; blood pressure, *n* = 7). A total of 49,799 participants were included in this research (Figure 1).

### Data collection

Demographic and lifestyle information, medical history, and current medication use were collected using a structured, study-specific questionnaire developed for the REACTION program and administered by trained staff under a standardized protocol across all participating centers. The questionnaire covered sociodemographic characteristics and lifestyle behaviors (including smoking and alcohol intake), self-reported prior physician diagnoses, and medication use (including lipid-lowering drugs). Details of the questionnaire and baseline data collection procedures have been described in the REACTION cohort profile [15]. Information on fatty liver, retinopathy, peripheral arterial disease, cardiovascular disease, stroke, and myocardial infarction was obtained from the baseline questionnaire based on self-reported prior physician diagnoses and current medication use. Diabetes was defined as meeting any of the following criteria: fasting blood glucose (FBG) ≥ 7.0 mmol/L (126 mg/dL) and/or 2-h plasma glucose during an oral glucose tolerance test (OGTT) ≥ 11.1 mmol/L (200 mg/dL), and/or glycated hemoglobin (HbA1c) ≥ 6.5% [16]. In addition, diabetes was also ascertained based on a self-reported prior physician diagnosis of diabetes or self-reported use of insulin or oral glucose-lowering medications, regardless of the current glycemic measurements. Physical measurements included height, body weight, waist circumference (WC), hip circumference (HC), and blood pressure.

Anthropometric measurements were performed by trained personnel with participants wearing light clothing and no shoes. Height and weight were measured using a calibrated stadiometer and scale, respectively. Body mass index (BMI) was calculated as weight in kilograms divided by height in meters squared (kg/m^2^). Systolic and diastolic blood pressure (SBP and DBP) were measured three times consecutively at 1-min intervals using an automated electronic device (OMRON Model HEM-752 FUZZY; Omron Co., Dalian, China) after the participants had rested for at least 5 min. The average of the three readings was used for analysis.

All participants fasted overnight for at least 10 h before blood sampling. Serum samples were processed within 2 h and shipped to the central laboratory at the Shanghai Institute of Endocrine and Metabolic Diseases, which is certified by the College of American Pathologists (CAP). Alanine aminotransferase (ALT), aspartate aminotransferase (AST), gamma-glutamyl transferase (GGT), fasting blood glucose (FBG), postprandial blood glucose (PBG), and serum creatinine (SCr) were measured using an automated analyzer (Cobas 8000 Modular Analyzer Series; Roche Diagnostics, Basel, Switzerland). Hemoglobin A1c (HbA1c) was measured by high-performance liquid chromatography (VARIANT II and D-10 Systems; Bio-Rad, Hercules, CA, USA). Lipid profiles, including total cholesterol (TC), low-density lipoprotein cholesterol (LDL-C), high-density lipoprotein cholesterol (HDL-C), and triglycerides (TG), were measured using an autoanalyzer (Abbott Laboratories, IL, USA). Urinary albumin was measured by immunonephelometry using Siemens BNII and BN ProSpec nephelometers (Siemens Healthcare Diagnostics, Marburg, Germany). Urinary creatinine was measured by the enzymatic method using the ADVIA Chemistry XPT System (Siemens Healthcare, Erlangen, Germany). The urine albumin-to-creatinine ratio (UACR) was calculated based on these measurements.

### Definition of variables

In this study, key indicators were calculated as follows. The UACR was determined from first-morning urine samples using the formula: UACR (mg/g) = urine albumin (mg/L)/urine creatinine (g/L). The AIP was defined as log_10_(TG/HDL‑C), with both TG and HDL‑C expressed in mmol/L. For data originally reported in mg/dL, unit conversions were applied as follows: TG (mmol/L) = TG (mg/dL) × 0.0113; HDL‑C (mmol/L) = HDL‑C (mg/dL) × 0.0259. Estimated glomerular filtration rate (eGFR) was calculated using the modified MDRD equation for Chinese participants: eGFR (mL/min/1.73 m^2^) = 186 × [SCr (mg/dL)/88.4] ^− 1.154^ × age ^− 0.203^ × (0.742 if female) × 1.233 (for Chinese population) [17].

According to KDIGO CKD guidelines, participants were categorized based on UACR as follows: normal (UACR < 30 mg/g) and albuminuria (UACR ≥ 30 mg/g) [18]. AIP values were divided into quartiles (Q1–Q4) based on the sample distribution: Q1 (lowest) to Q4 (highest). Quartiles were used to compare characteristics across groups and to perform trend tests, with Q1 serving as the reference. In regression models, AIP was also treated as a continuous variable to enhance the robustness of statistical estimates.

### Statistical methods

All statistical analyses were performed using SPSS 30.0 (IBM, Chicago, IL, USA). Continuous variables were tested for normality and, due to skewed distributions, are presented as median (interquartile range). Categorical variables are expressed as numbers and percentages. Group comparisons for continuous variables were conducted using the Kruskal–Wallis test (across AIP quartiles) or the Mann–Whitney U test (between UACR groups), while the chi-square test was used for categorical variables. Variables with missing values (each with a missing rate <20%) were handled by multiple imputation (*m* = 5).

Using the first quartile of AIP (Q1) as the reference group, a logistic regression model (Model 1, unadjusted) was constructed to examine the association between AIP and albuminuria. To rigorously assess this relationship while controlling for potential confounders, we built a directed acyclic graph (DAG) based on prior literature and clinical knowledge to identify potential confounders in the AIP–albuminuria relationship and to determine the minimally sufficient adjustment set (MSAS). Based on the DAG-identified MSAS (Supplementary Figure 1), a multivariable logistic regression model (Model 2, adjusted) was built, including covariates such as age, sex, lifestyle factors (smoking and alcohol consumption), BMI, diabetes, hypertension, and eGFR, to block potential confounding pathways and obtain more robust effect estimates for the AIP–albuminuria association. Results are presented as odds ratios (ORs) with 95% confidence intervals (CIs). Multicollinearity among covariates in Model 2 was assessed using variance inflation factors (VIF), while all VIF values were <5 (Supplementary Table 2).

Stratified analyses were performed to further explore the relationship between AIP and UACR across subgroups defined by age (<60/≥60 years), sex, BMI (<24/≥24 kg/m^2^), lipid-lowering drug use (yes/no), and status of diabetes, hypertension, and hyperlipidemia. Age was stratified at 60 years, a commonly used cut-point to define older adults in China [19,20]. BMI <24 vs ≥24 kg/m^2^ was used to distinguish normal weight from overweight/obesity according to Chinese criteria (overweight: BMI ≥24.0 kg/m^2^; obesity: BMI ≥28.0 kg/m^2^) [21].

All tests were two-sided, and a *p* value < 0.05 was considered statistically significant.

## Results

### Characteristics of study population

This study included 49,799 participants (15,754 men and 34,045 women). The median age of the population was 57.41 years (IQR 52.01–63.73). As shown in Table 1, participants with UACR ≥30 mg/g were older and had a significantly higher prevalence of diabetes and fatty liver disease. Compared with those with normal UACR, individuals with albuminuria exhibited significantly higher levels of TG, ALT, AST, GGT, FBG, PBG, HbA1c, fasting insulin, waist and hip circumferences, systolic blood pressure (SBP), diastolic blood pressure (DBP), and BMI, whereas HDL-C and eGFR were significantly lower (all *p* < 0.001). Comparisons across AIP quartiles further showed that, relative to Q1, participants in Q2–Q4 had significantly higher values for age, male proportion, and displayed higher BMI, blood pressure, glycemic indices, lipid parameters, and liver enzyme levels, along with a higher prevalence of metabolic disorders (Supplementary Table 1). Conversely, they had significantly lower levels of HDL-C and estimated glomerular filter rate (eGFR). Also, along with rising AIP quartiles, UACR showed the same elevating trend.

**Table 1.** Characteristics of study population by UACR dichotomy.

Variable	UACR < 30 mg/g	UACR ≥ 30 mg/g	
	(*n* = 43,564)	(*n* = 6235)	*p* value
Age, y	57.02 (51.77, 62.95)	60.83 (54.15, 68.82)	<0.001
Male, *n* (%)	14,070(32.3)	1684 (27.0)	<0.001
Current status, *n* (%)	–	–	–
Drinking	2988 (6.9)	357 (5.7)	<0.001
Smoking	5145 (11.8)	660 (10.6)	<0.001
Anamnesis, *n* (%)	–	–	–
Myocardial Infarction	144 (0.3)	38 (0.6)	<0.001
Stroke	462 (1.1)	117 (1.9)	<0.001
Cardiovascular Disease	1413 (3.2)	354 (5.7)	<0.001
Hypertension	7196 (16.5)	2041 (32.7)	<0.001
Lower Extremity Arterial Disease	49 (0.1)	16 (0.3)	0.004
Retinopathy	241 (0.6)	67 (1.1)	<0.001
Hyperlipidemia	3340 (7.7)	636 (10.2)	<0.001
Fatty Liver Disease	3052 (7.0)	565 (9.1)	<0.001
Diabetes Mellitus	3802 (8.7)	1253 (20.1)	<0.001
Lipid-lowering drugs use, *n* (%)	386 (0.9)	58 (0.9)	ns
BMI, kg/m^2^	24.20 (22.07, 26.46)	24.70 (22.45, 27.20)	<0.001
Waist Circumference, cm	85.00 (79.00, 92.00)	87.50 (80.00, 94.30)	<0.001
Hip Circumference, cm	96.00 (91.40, 101.00)	98.00 (93.00, 103.00)	<0.001
SBP, mmHg	128.67 (116.67, 142.67)	138.00 (122.33, 154.00)	<0.001
DBP, mmHg	76.67 (70.00, 83.67)	78.83 (71.33, 87.00)	<0.001
LDL-C, mmol/L	2.97 (2.39, 3.58)	2.86 (2.28, 3.46)	<0.001
HDL-C, mmol/L	1.31 (1.10, 1.54)	1.24 (1.05, 1.47)	<0.001
CHOL, mmol/L	5.09 (4.36, 5.83)	5.00 (4.27, 5.75)	0.010
TG, mmol/L	1.33 (0.96, 1.92)	1.56 (1.10, 2.25)	<0.001
ALT, U/L	14.00 (11.00, 20.00)	15.00 (11.00, 22.00)	<0.001
AST, U/L	20.00 (17.00, 24.00)	21.00 (17.00, 26.00)	<0.001
GGT, U/L	21.00 (15.00, 31.00)	22.00 (15.00, 35.00)	<0.001
UACR, mg/g	8.00 (4.73, 13.68)	48.22 (36.33, 80.46)	<0.001
CREA, μmol/L	65.50 (59.50, 73.10)	66.60 (59.90, 76.10)	<0.001
eGFR, mL/min/1.73m²	114.25 (102.19, 128.07)	109.14 (95.94, 123.87)	<0.001
FBG, mmol/L	5.52 (5.11, 6.10)	5.80 (5.22, 6.96)	<0.001
PBG, mmol/L	7.25 (5.96, 9.40)	8.51 (6.60, 12.30)	<0.001
HbA1c, (%)	5.80 (5.60, 6.20)	6.10 (5.70, 6.70)	<0.001
Fasting Insulin, uU/mL	7.30 (5.30, 10.00)	8.10 (5.80, 11.50)	<0.001

All continuous data in this table were non-normally distributed. Therefore, they are presented as median (interquartile range) for group comparisons. Categorical variables are presented as numbers (percentages).

BMI: body mass index; SBP: systolic blood pressure; DBP: diastolic blood pressure; LDL-C: low-density lipoprotein cholesterol; HDL-C: high-density lipoprotein cholesterol; CHOL: cholesterol; TG: triglycerides; ALT: alanine aminotransferase; AST: aspartate aminotransferase; GGT: gamma-glutamyl transferase; UACR: urine albumin-to-creatinine ratio; CREA: serum creatinine; eGFR: estimated glomerular filtration rate; FBG: fasting blood glucose; PBG: postprandial blood glucose; HbA1c: glycosylated hemoglobin, type A1c.

### Correlation between AIP and blood lipid indicators

Spearman correlation analysis revealed that AIP showed an almost negligible correlation with LDL-C (ρ = −0.009, *p* < 0.001) and no significant association with TC (Table 2).

**Table 2.** Corrections between AIP and other lipid parameters.

ρ	AIP	LDL-C	CHOL
AIP	–	−0.009 (*p* < 0.001)	0.043 (*p* = 1)
LDL-C	–	–	0.891 (*p* < 0.001)
CHOL	–	–	–

AIP: atherogenic index of plasma; LDL-C: low-density lipoprotein cholesterol; CHOL: total cholesterol.

### Correlation between AIP and UACR

Table 3 presents the odds ratios (OR) and 95% confidence intervals (CI) for the association between AIP quartiles and albuminuria. Both Model 1 (unadjusted) and Model 2 (adjusted) indicated significant association between AIP and albuminuria. After adjustment for age, sex, lifestyle factors (smoking and alcohol consumption), BMI, diabetes, hypertension, and eGFR, the association between AIP and albuminuria remained significant. Compared with the lowest AIP quartile, the adjusted ORs for albuminuria in the second, third, and fourth quartiles were 1.304 (95% CI: 1.196, 1.422), 1.475 (95% CI: 1.355, 1.607), and 1.815 (95% CI: 1.668, 1.975), respectively, indicating a significant positive dose-response relationship. Furthermore, this positive association remained significant when AIP was analyzed as a continuous variable (OR: 2.149, 95% CI:1.951–2.367, *p* < 0.001).

**Table 3.** Association between AIP quartile and UACR in Chinese urban adults.

	Model 1		Model 2	
	OR (95% CI)	*p* value	OR (95% CI)	*p* value
Q1	Reference	–	Reference	–
Q2	1.443 (1.325, 1.570)	<0.001	1.304 (1.196, 1.422)	<0.001
Q3	1.771 (1.632, 1.922)	<0.001	1.475 (1.355, 1.607)	<0.001
Q4	2.279 (2.105, 2.467)	<0.001	1.815 (1.668, 1.975)	<0.001
*p* for Trend	–	<0.001	–	<0.001
AIP Continuous	2.700 (2.472, 2.948)	<0.001	2.149 (1.951, 2.367)	<0.001

Model 1: unadjusted.

Model 2: adjusted for age, sex, BMI, smoking status, drinking status, history of hypertension, history of diabetes, and eGFR.

### Subgroup analysis

To further explore the relationship between AIP and albuminuria, we performed a stratified analysis by age, sex, BMI, and history of hypertension, hyperlipidemia, lipid-lowering drug use, and diabetes mellitus (Table 4). Interaction analysis indicated that age and lipid-lowering drug use were significant effect modifiers of the AIP-albuminuria association (*p* for interaction = 0.046 and 0.017, respectively). In contrast, no significant interaction was observed for sex, BMI, hypertension, or hyperlipidemia (all *p* for interaction > 0.05). The association between AIP and albuminuria was significantly stronger in participants younger than 60 years (*p* for trend < 0.001) than in those aged 60 years or older (*p* for trend = 0.028). A significant positive association between AIP and albuminuria was observed in participants not taking lipid-lowering drugs (*p* for trend < 0.001), whereas no significant association was found in those who were (*p* for trend = 0.422). In summary, the AIP-albuminuria correlation was strongest in younger individuals (age < 60 years) not using lipid-lowering drugs.

**Table 4.** Subgroup analyses of association between WWI tertile and T2DM risk in Chinese urban adults.

Variable	AIP quartiles					
	Q1 OR (95%CI)	Q2 OR (95%CI)	Q3 OR (95%CI)	Q4 OR (95%CI)	*p* for trend	*p* for interaction
Age, years	–	–	–	–	–	0.046
<60	1	1.325 (1.162, 1.511)***	1.518 (1.312, 1.756)***	1.750 (1.460, 2.098)***	<0.001	–
≥60	1	1.294 (1.125, 1.489)***	1.338 (1.145, 1.565)***	1.286 (1.057, 1.566)*	0.028	–
Lipid-lowering drug use	–	–	–	–	–	0.017
No	1	1.332 (1.210, 1.466)***	1.447 (1.300, 1.610)***	1.565 (1.370, 1.787)***	<0.001	–
Yes	1	0.329 (0.078, 1.385)^ns^	1.524 (0.425, 5.460)^ns^	0.431 (0.092, 2.027)^ns^	0.422	–
Sex	–	–	–	–	–	>0.05
BMI	–	–	–	–	–	>0.05
Hypertension	–	–	–	–	–	>0.05
Hyperlipidemia	–	–	–	–	–	>0.05
Diabetes Mellitus	–	–	–	–	–	>0.05

*p value < 0.05; **p value < 0.01; ***p value < 0.001; ^ns^*p* value >0.05.

Model adjusted for age, sex, BMI, smoking status, drinking status, history of hypertension, history of hyperlipidemia, history of lipid-lowering drug use, creatinine, and eGFR, SBP, DBP, waist circumference, hip circumference, LDL-C, CHOL, TG, ALT, AST, GGT, FBG, PBG, HbA1c, fasting insulin, history of fatty liver disease, history of retinopathy, history of LEAD, history of CVD, history of stoke, history of myocardial infarction, history of diabetes.

BMI: body mass index; eGFR: estimated glomerular filtration rate; SBP: systolic blood pressure; DBP: diastolic blood pressure; LDL-C: low-density lipoprotein cholesterol; CHOL: cholesterol; TG: triglycerides; ALT: alanine aminotransferase; AST: aspartate aminotransferase; GGT: gamma-glutamyl transferase; FBG: fasting blood glucose; PBG: postprandial blood glucose; HbA1c: glycosylated hemoglobin, type A1c; LEAD: lower extremity arterial disease; CVD: cardiovascular diseases.

## Discussion

AIP is calculated as the logarithm of the ratio of TG to HDL-C and is considered as a valuable indicator which can comprehensively reflect the risk of atherosclerosis and dyslipidemia. This study found that a higher AIP level was significantly positively correlated with albuminuria (UACR ≥ 30 mg/g) in a Chinese urban population, even after adjusting for various confounding factors. In subgroup analyses, this positive correlation persisted, particularly among participants younger than 60 years and those not using lipid-lowering drugs.

A cross-sectional study utilizing data from two national databases, the US National Health and Nutrition Examination Survey (NHANES) and the Korean National Health and Nutrition Examination Survey (KNHANES), found that higher AIP was significantly associated with albuminuria [22]. Similarly, a retrospective study of 600 adolescents in Saudi Arabia who suffered from type 1 diabetes mellitus found that AIP was significantly positively correlated with UACR^13^. In a cross-sectional study of Chinese adults in Shaanxi, China, Yuan et al. found that a higher AIP was significantly correlated with elevated UACR and reduced eGFR, suggesting that AIP may serve as an independent risk factor for early renal injury [23]. Additionally, a study of 335 patients with newly diagnosed diabetes mellitus by Qi et al. identified AIP as an independent risk factor for microalbuminuria, with the area under the ROC curve being approximately 0.77 [24]. Our findings are consistent with the previous studies. Furthermore, based on a larger sample from a general urban population, our results further confirm the positive correlation between a higher AIP and elevated UACR.

An elevated AIP reflects a state of dyslipidemia characterized by high TG and low HDL-C levels [11], a pattern consistent with our findings. The lipotoxicity of TG and TG-rich lipoproteins to the kidney has been widely documented in numerous studies. TG can accumulate as lipid droplets in podocytes and proximal tubules, inducing mitochondrial dysfunction and increased ROS production. This subsequently impairs the glomerular filtration barrier and tubular function, ultimately leading to albuminuria [25–27]. Studies have confirmed that high TG is an independent risk factor for poor renal outcomes [28,29]. Furthermore, there are studies suggesting that HDL-C reduction or dysfunction accelerates renal injury by impairing cellular cholesterol efflux [26,30]. Excess cholesterol can also induce mitochondrial dysfunction, ROS generation, and inflammation, thereby impairing the glomerular filtration barrier and tubular function, and ultimately leading to albuminuria and renal impairment [31–33]. Moreover, reduced or dysfunctional HDL may lead to decreased levels of protective components, such as Apo M, Apo E, and Apo L1, thereby compromising systemic anti-inflammatory and antioxidant capacities [34]. AIP can serve as a simple surrogate indicator for small dense LDL (sdLDL) [35,36]. Due to its small particle diameter and high susceptibility to oxidation, sdLDL can permeate the glomerular endothelium and accumulate in the mesangium. This process induces endothelial inflammation and dysfunction, triggering the release of vasoactive substances and ultimately increasing albumin leakage [26,37,38]. Furthermore, AIP-related dyslipidemia is closely associated with insulin resistance and chronic low-grade inflammation [10,39,40]. Insulin resistance exacerbates oxidative stress *via* activation of the renin-angiotensin-aldosterone system (RAAS), whereas chronic inflammation directly compromises the integrity of the renal filtration barrier. Together, these factors can form a vicious cycle with dyslipidemia, collectively relating to an elevated risk of albuminuria. This interplay represents an important underlying mechanism for the positive correlation between AIP and UACR [41–43].

Our stratified analysis revealed a significant positive association between AIP and UACR in participants younger than 60 years, which was markedly attenuated in older adults, indicating a significant effect modification by age. This finding suggests that the association between AIP and UACR may be stronger in younger populations. The weakened correlation in the elderly may be attributed to two factors: first, the multifactorial pathogenesis of renal impairment in aging, including inflammaging, vascular stiffness, and chronic endothelial dysfunction, which may overshadow the relative contribution of pure lipotoxicity [44,45]; second, the more prevalent use of lipid-lowering drugs in older adults, which ameliorates the very lipid parameters (e.g., TG, HDL-C, sdLDL) that AIP reflects, thereby diluting its statistical association with albuminuria. This study also found a more pronounced dose-response relationship between AIP and UACR in non-users of lipid-lowering drugs, which aligns with the findings above. In summary, the association between AIP and UACR appears to be more pronounced in middle-aged adults who are not using lipid-lowering medication. However, future large-scale, prospective studies with repeated UACR measurements are needed to confirm temporality and evaluate potential clinical utility.

Our findings establish AIP as a handy, reliable, inexpensive, and reproducible indicator that is independently associated with UACR. This association was more pronounced among individuals younger than 60 years and those not using lipid-lowering medications, suggesting a closer link between atherogenic dyslipidemia and albuminuria in these subgroups. Overall, AIP may serve as a metabolic marker related to albuminuria and provides a basis for further evaluation of its clinical relevance in larger populations and longitudinal studies. The key strength of our study is that it is the first large-scale, cross-sectional investigation conducted in a community-based Chinese population. The inclusion of participants from multiple centers across China enhances the representativeness and generalizability of our findings. However, this study has several limitations. First, its cross-sectional design can only reveal an association between AIP and UACR and cannot establish causality. Second, owing to the use of a single first-morning urine measurement for UACR without longitudinal validation, this study cannot evaluate the ability of AIP to predict long-term renal damage. Third, although the multivariable models adjusted for multiple potential confounders identified by the DAG, we cannot fully rule out residual confounding, which might lead to biased estimates. Prospective longitudinal studies are needed to clarify the biological mechanisms underlying this correlation and to evaluate the potential of AIP-lowering interventions.

## Conclusion

This cross-sectional study, based on a Chinese urban adult population, found a significant positive association between AIP and albuminuria (elevated UACR), and the association was more pronounced among individuals younger than 60 years and those not using lipid-lowering medications. As an accessible and low-cost composite index derived from routine lipid parameters, AIP may provide complementary metabolic information related to albuminuria, and may help more comprehensively characterize metabolic profiles when interpreted alongside UACR and eGFR. Future prospective cohort studies with repeated UACR measurements are warranted to further validate the stability and temporality of this association and to elucidate potential causal mechanisms and underlying biological pathways.

## Supplementary Material

Supplementary Figure1.jpeg

Supplementary Table 1.docx

Supplementary Table 2.docx

## Data Availability

The data that support the findings of this study are available from the corresponding authors upon reasonable request.
