# Supplementary figures and images for: Association of atherogenic index of plasma with urine albumin-to-creatinine ratio in Chinese urban adults: a cross-sectional study
Source: Ren Fail. 2026 Jun 1;48(1):2657102. doi: 10.1080/0886022X.2026.2657102 (PMC13231815; doi:10.1080/0886022X.2026.2657102)

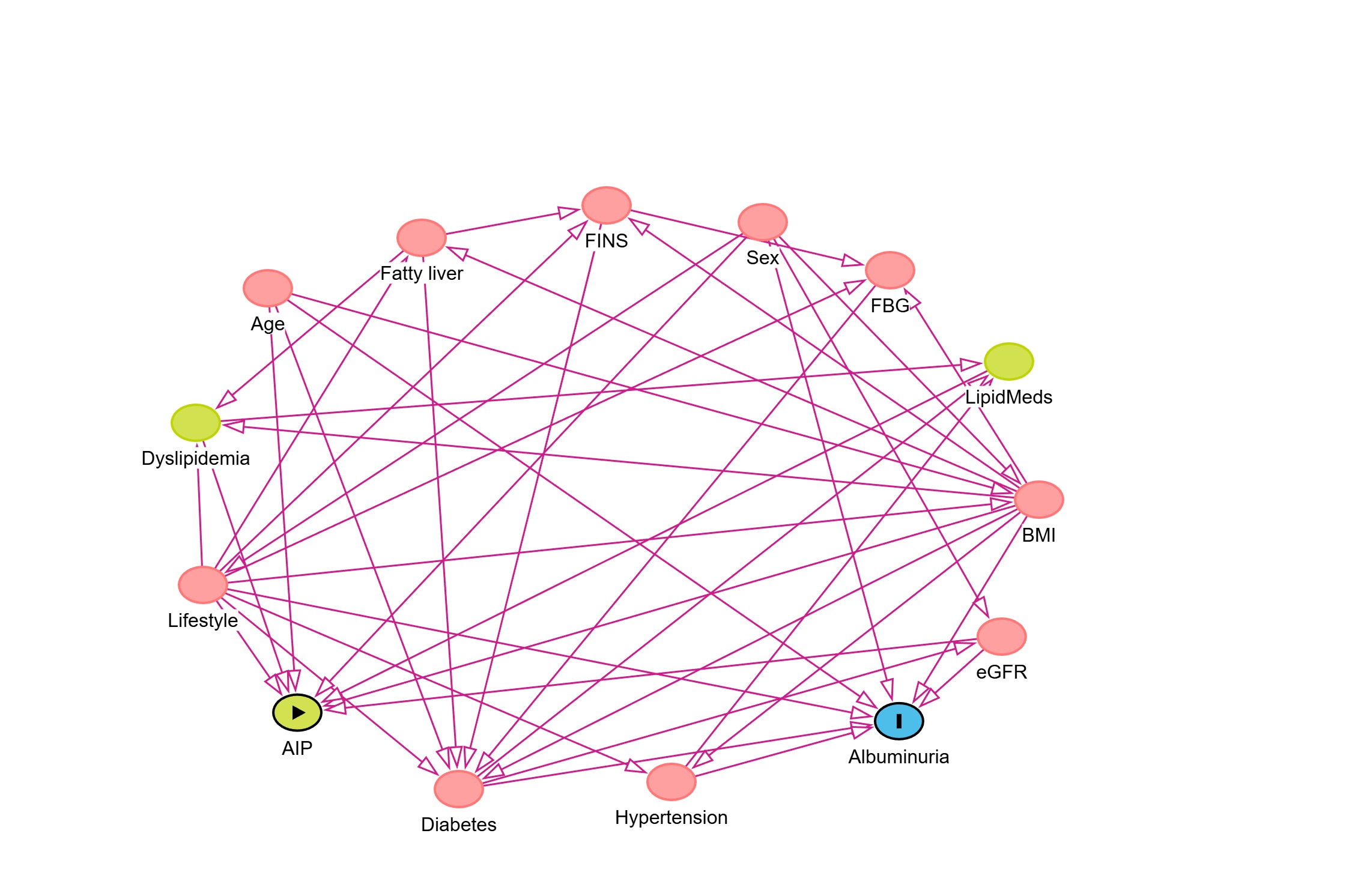

Supplement: Supplementary Figure1.jpeg [file IRNF_A_2657102_SM4777.jpeg]
