# Supplementary material for: Association of atherogenic index of plasma with urine albumin-to-creatinine ratio in Chinese urban adults: a cross-sectional study
Source: Ren Fail. 2026 Jun 1;48(1):2657102. doi: 10.1080/0886022X.2026.2657102 (PMC13231815; doi:10.1080/0886022X.2026.2657102)
Supplement: Supplementary Table 1.docx [file IRNF_A_2657102_SM4776.docx]

Supplementary Table 1. Characteristics of study population by quartiles of AIP

| Variable |  | Quartiles of AIP | | | |  |
| --- | --- | --- | --- | --- | --- | --- |
|  | Total | Q1 | Q2 | Q3 | Q4 | P-Value |
|  | (n=49799) | (n=12454) | (n=12449) | (n=12442) | (n=12454) |  |
| Age, y | 57.41 (52.01, 63.73) | 55.76 (50.33, 61.82) | 57.23 (52.02, 63.71) | 58.19 (53.04, 64.60) | 58.31 (52.98, 64.43) | <0.001 |
| Male, n (%) | 15754 (31.6) | 3102 (24.9) | 3593 (28.9) | 4102 (33.0) | 4957 (39.8) | <0.001 |
| Current status, n (%) | - | - | - | - | - | - |
| Drinking | 3345 (6.7) | 823 (6.6) | 758 (6.1) | 768 (6.2) | 996 (8.0) | <0.001 |
| Smoking | 5805 (11.7) | 1067 (8.6) | 1271 (10.2) | 1472 (11.8) | 1995 (16.0) | <0.001 |
| Anamnesis, n (%) | - | - | - | - | - | - |
| Myocardial Infarction | 182 (0.4) | 21 (0.2) | 29 (0.2) | 61 (0.5) | 71 (0.6) | <0.001 |
| Stroke | 579 (1.2) | 83 (0.7) | 133 (1.1) | 169 (1.4) | 194 (1.6) | <0.001 |
| Cardiovascular Disease | 1767 (3.5) | 254 (2.0) | 435 (3.5) | 513 (4.1) | 565 (4.5) | <0.001 |
| Hypertension | 9237 (18.5) | 1312 (10.5) | 1951 (15.7) | 2659 (21.4) | 3315 (26.6) | <0.001 |
| Lower Extremity Arterial Disease | 65 (0.1) | 12 (0.1) | 20 (0.2) | 16 (0.1) | 17 (0.1) | ns |
| Retinopathy | 308 (0.6) | 48 (0.4) | 71 (0.6) | 86 (0.7) | 103 (0.8) | <0.001 |
| Hyperlipidemia | 3976 (8.0) | 443 (3.6) | 698 (5.6) | 1059 (8.5) | 1776 (14.3) | <0.001 |
| Fatty Liver Disease | 3617 (7.3) | 337 (2.7) | 615 (4.9) | 1075 (8.6) | 1590 (12.8) | <0.001 |
| Diabetes Mellitus | 5055 (10.2) | 779 (6.3) | 1055 (8.5) | 1346 (10.8) | 1875 (15.1) | <0.001 |
| Lipid-lowering drugs use, n (%) | 444 (0.9) | 75 (0.6) | 95 (0.8) | 120 (1.0) | 154 (1.2) | <0.001 |
| BMI, kg/m2 | 24.24 (22.12, 26.56) | 22.72 (20.74, 24.94) | 23.81 (21.79, 26.07) | 24.76 (22.77, 27.01) | 25.44 (23.53, 27.56) | <0.001 |
| Waist Circumference, cm | 85.20 (79.00, 92.00) | 81.00 (74.20, 88.00) | 84.00 (78.00, 90.35) | 87.00 (81.00, 94.00) | 89.00 (83.00, 95.00) | <0.001 |
| Hip Circumference, cm | 96.00 (92.00, 101.00) | 94.00 (89.00, 99.00) | 96.00 (91.00, 100.00) | 97.00 (93.00, 102.00) | 98.3 (94.00, 103.00) | <0.001 |
| SBP, mmHg | 129.67 (117.33, 144.00) | 125.00 (113.00, 140.00) | 128.67 (116.33, 142.67) | 131.00 (119.00, 145.67) | 133.33 (121.00, 147.33) | <0.001 |
| DBP, mmHg | 76.67 (70.00, 84.00) | 74.00 (67.33, 81.33) | 76.00 (69.33, 83.33) | 77.67 (71.00, 84.67) | 79.33 (72.67, 86.33) | <0.001 |
| LDL-C, mmol/L | 2.95 (2.37, 3.56) | 2.85 (2.31, 3.42) | 3.05 (2.48, 3.66) | 3.13 (2.53, 3.72) | 2.79 (2.21, 3.40) | <0.001 |
| HDL-C, mmol/L | 1.30 (1.09, 1.53) | 1.63 (1.42, 1.85) | 1.38 (1.21, 1.55) | 1.23 (1.08, 1.39) | 1.06 (0.93, 1.21) | <0.001 |
| CHOL, mmol/L | 5.08 (4.34, 5.82) | 5.03 (4.35, 5.72) | 5.05 (4.33, 5.80) | 5.12 (4.37, 5.88) | 5.11 (4.33, 5.88) | <0.001 |
| TG, mmol/L | 1.36 (0.97, 1.97) | 0.80 (0.67, 0.94) | 1.16 (1.01, 1.33) | 1.60 (1.38, 1.83) | 2.56 (2.11, 3.31) | <0.001 |
| ALT, U/L | 15.00 (11.00, 21.00) | 13.00 (10.00, 18.00) | 14.00 (10.00, 19.00) | 15.00 (11.00, 21.00) | 17.00 (12.00, 25.00) | <0.001 |
| AST, U/L | 20.00 (17.00, 25.00) | 20.00 (17.00, 24.00) | 20.00 (17.00, 24.00) | 20.00 (17.00, 25.00) | 21.00 (17.00, 26.00) | <0.001 |
| GGT, U/L | 21.00 (15.00, 32.00) | 17.00 (13.00, 24.00) | 19.00 (14.00, 28.00) | 22.00 (16.00, 33.00) | 27.00 (19.00, 41.00) | <0.001 |
| UACR, mg/g | 9.28 (5.19, 18.78) | 7.55 (4.44, 14.27) | 8.89 (5.02, 17.74) | 9.96 (5.59, 20.13) | 11.40 (5.99, 22.89) | <0.001 |
| CREA, μmol/L | 65.7 (59.5, 73.4) | 63.70 (58.30, 70.60) | 65.00 (59.20, 72.40) | 66.10 (59.90, 74.10) | 67.90 (61.00, 77.80) | <0.001 |
| eGFR, mL/min/1.73m² | 113.64 (101.32, 127.52) | 116.11 (104.21, 129.25) | 113.98 (102.12, 127.87) | 112.68 (100.69, 126.73) | 111.65 (98.58, 126.24) | <0.001 |
| FBG, mmol/L | 5.55 (5.13, 6.18) | 5.37 (5.00, 5.81) | 5.49 (5.10, 6.01) | 5.60 (5.18, 6.23) | 5.81 (5.30, 6.70) | <0.001 |
| PBG, mmol/L | 7.39 (6.01, 9.70) | 6.45 (5.45, 7.97) | 7.10 (5.90, 9.01) | 7.69 (6.28, 10.10) | 8.60 (6.85, 11.53) | <0.001 |
| HbA1c, (%) | 5.90 (5.60, 6.20) | 5.70 (5.50, 6.00) | 5.80 (5.60, 6.13) | 5.90 (5.60, 6.30) | 6.00 (5.70, 6.50) | <0.001 |
| Fasting Insulin, uU/mL | 7.40 (5.30, 10.20) | 5.70 (4.20, 7.70) | 6.80 (5.10, 9.20) | 8.00 (5.90, 10.90) | 9.40 (7.00, 12.50) | <0.001 |

All continuous data in this table were non-normally distributed. Therefore, they are presented as median (interquartile range) for group comparisons. Categorical variables are presented as numbers (percentages).

Abbreviations: BMI, body mass index; SBP, systolic blood pressure; DBP, diastolic blood pressure; LDL-C, low-density lipoprotein cholesterol; HDL-C, high-density lipoprotein cholesterol; CHOL, cholesterol; TG, triglycerides; ALT, alanine aminotransferase; AST, aspartate aminotransferase; GGT, gamma-glutamyl transferase; UACR, urine albumin-to-creatinine ratio; CREA, serum creatinine; eGFR, estimated glomerular filtration rate; FBG, fasting blood glucose; PBG, postprandial blood glucose; HbA1c, glycosylated hemoglobin, type A1c.
