# Supplementary material for: Association of atherogenic index of plasma with urine albumin-to-creatinine ratio in Chinese urban adults: a cross-sectional study
Source: Ren Fail. 2026 Jun 1;48(1):2657102. doi: 10.1080/0886022X.2026.2657102 (PMC13231815; doi:10.1080/0886022X.2026.2657102)
Supplement: Supplementary Table 2.docx [file IRNF_A_2657102_SM4775.docx]

| Variables | Variance Inflation Factor (VIF) | | | | | |
| --- | --- | --- | --- | --- | --- | --- |
|  | Raw data | Imputation 1 | Imputation 2 | Imputation 3 | Imputation 4 | Imputation 5 |
| AIP | 1.129 | 1.126 | 1.127 | 1.126 | 1.126 | 1.126 |
| Gender | 1.586 | 1.573 | 1.570 | 1.573 | 1.574 | 1.573 |
| Age | 1.240 | 1.237 | 1.237 | 1.238 | 1.237 | 1.237 |
| BMI | 1.112 | 1.109 | 1.110 | 1.110 | 1.110 | 1.109 |
| Smoking | 1.467 | 1.461 | 1.459 | 1.460 | 1.462 | 1.460 |
| Drinking | 1.324 | 1.321 | 1.319 | 1.319 | 1.320 | 1.319 |
| Hypertension | 1.128 | 1.125 | 1.125 | 1.125 | 1.124 | 1.125 |
| Diabetes Mellitus | 1.054 | 1.054 | 1.054 | 1.054 | 1.054 | 1.054 |
| eGFR | 1.162 | 1.160 | 1.160 | 1.160 | 1.160 | 1.160 |

Supplementary Table 2

Abbreviations: AIP, atherogenic index of plasma; BMI, body mass index; eGFR, estimated glomerular filtration rate.
